# Central Airway Carcinoid Tumorlets Following Resection of a Typical Carcinoid Tumor

**DOI:** 10.3390/diagnostics15131651

**Published:** 2025-06-28

**Authors:** Kyungsoo Bae, Kyung Nyeo Jeon, I Re Heo, Hyo Jung An, Dae Hyun Song

**Affiliations:** 1 Department of Radiology, Institute of Medical Science, School of Medicine, Gyeongsang National University, Jinju 52727, Republic of Korea; ksbae@gnu.ac.kr; 2 Department of Radiology, Gyeongsang National University Changwon Hospital, Changwon 51472, Republic of Korea; 3 Department of Internal Medicine, School of Medicine, Gyeongsang National University, Gyeongsang National University Changwon Hospital, Changwon 51472, Republic of Korea; h2hawk@naver.com; 4 Department of Pathology, School of Medicine, Gyeongsang National University, Gyeongsang National University Changwon Hospital, Changwon 51472, Republic of Korea; ariel2020@naver.com (H.J.A.); golgy@hanmail.net (D.H.S.)

**Keywords:** bronchi, carcinoid tumor, tumorlets, neuroendocrine tumors, neuroendocrine cells, multiple primary neoplasms

## Abstract

Pulmonary neuroendocrine proliferations and neoplasms represent a broad spectrum of diseases, ranging from neuroendocrine cell hyperplasia and tumorlets to carcinoid tumors. Carcinoid tumorlets are most commonly located in the peripheral airways and are often incidentally detected as pulmonary micronodules on chest CT. We report the radiological, bronchoscopic, and pathological findings of a case of carcinoid tumorlets presenting as endobronchial nodules in the left main bronchus. The patient had previously undergone a left lower lobectomy five years earlier for a typical carcinoid tumor. Follow-up imaging revealed new endobronchial nodules, which were subsequently confirmed as carcinoid tumorlets through histopathologic analysis. This case highlights the rare presentation of carcinoid tumorlets in the central airways, emphasizing the importance of recognizing their potential for late recurrence and atypical localization. It underscores the necessity for physicians to be aware that pulmonary neuroendocrine tumors can recur over the long term and may present in a multicentric fashion within the disease spectrum.

**Figure 1:**
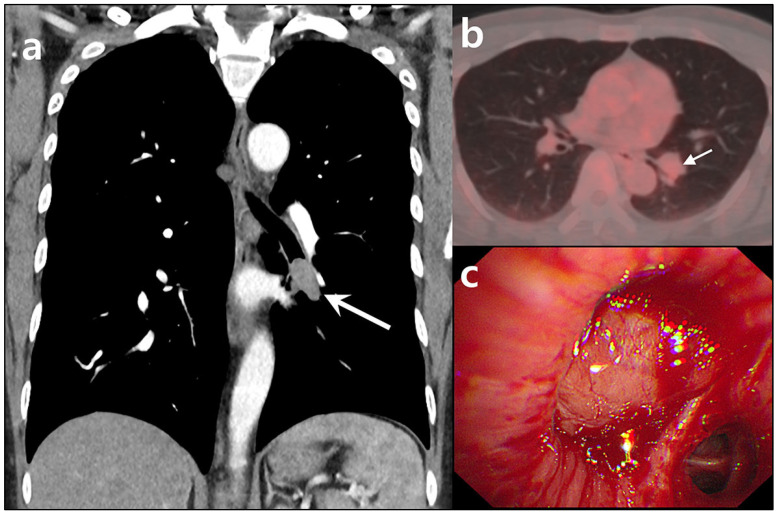
CT, PET/CT, and bronchoscopic images of a typical carcinoid tumor in the left lower lobe in a 40-year-old man. The patient reported experiencing intermittent blood-tinged sputum, which had started three months before presentation. He had a 20 pack-year smoking history and was on medication for hypertension. (**a**) Chest CT revealed a well-enhancing nodule measuring approximately 2.5 cm, located in the left lower lobe bronchus (arrow). (**b**) FDG-PET/CT showed the lesion to be mildly hypermetabolic, with an SUVmax of 2.6 (arrow). (**c**) Bronchoscopy identified a hypervascular, polypoid lesion obstructing the left lower lobe bronchus. A bronchoscopic biopsy indicated a carcinoid tumor. The patient subsequently underwent video-assisted thoracoscopic surgery (VATS) for a left lower lobectomy.

**Figure 2:**
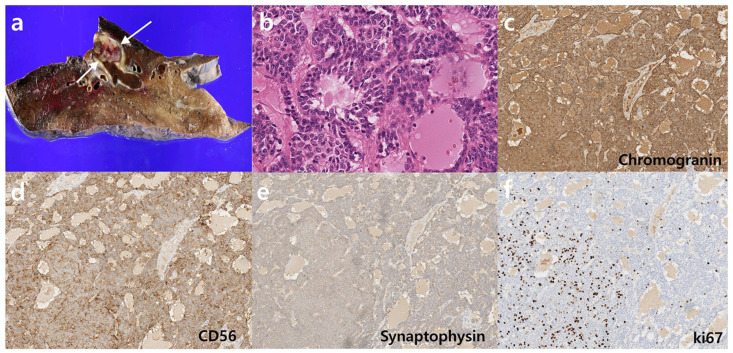
Gross and microscopic findings of a typical carcinoid tumor. (**a**) Gross examination of the left lower lobectomy specimen showed a well-demarcated 2.5 cm × 1.0 cm nodule (arrows) within the left lower lobar bronchus. (**b**) Microscopic examination (× 200) revealed neuroendocrine cell proliferation with inconspicuous, round, bland-looking nuclei, and no evidence of necrosis. There was 1 mitosis per 2 mm^2^. (**c**–**e**) Immunohistochemical staining was positive for chromogranin, CD56, and synaptophysin. (**f**) The Ki-67 index was less than 5%. The lesion was ultimately diagnosed as a typical carcinoid tumor. No lymph node metastasis was observed. The patient remained stable with annual CT follow-ups. However, in the fifth-year post-operation, abnormal findings were detected on chest CT.

**Figure 3:**
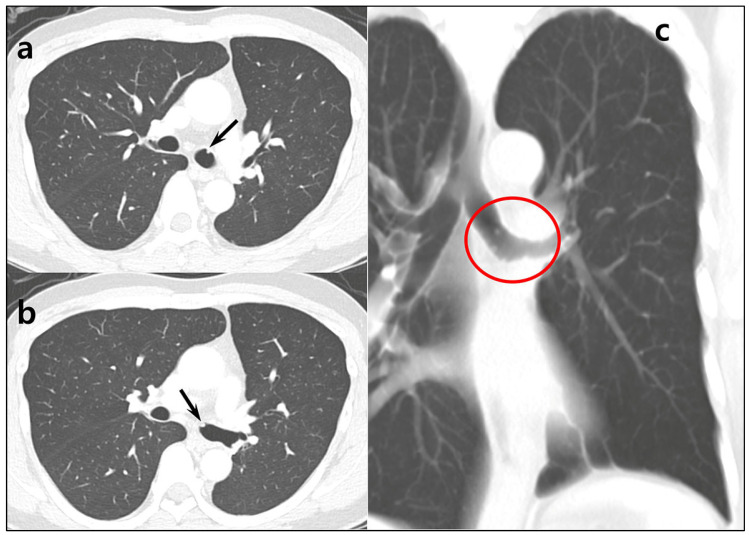
Follow-up CT images obtained in the fifth-year post-operation. (**a**,**b**) Chest CT revealed several tiny nodular densities in the left main bronchus (arrows). Although the possibility of bronchial secretions was initially considered, the lesions persisted on a 3-month follow-up CT. (**c**) A coronal thick slab reformatted image provided a clearer view of multiple nodular lesions, each measuring less than 5 mm (red circle).

**Figure 4:**
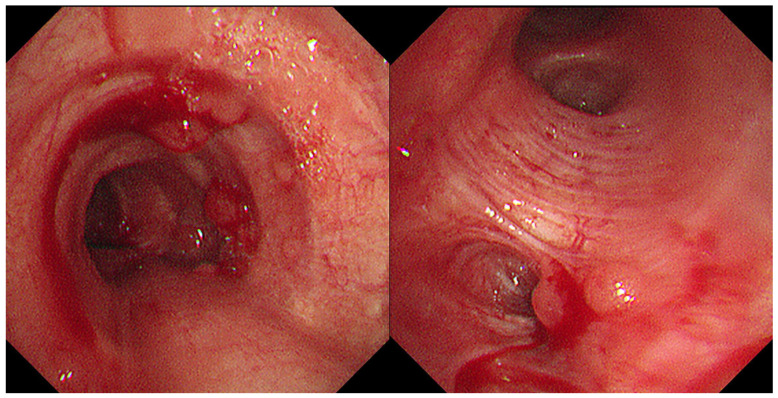
Bronchoscopy confirmed multiple polypoid lesions in the left main bronchus, each measuring less than 5 mm.

**Figure 5:**
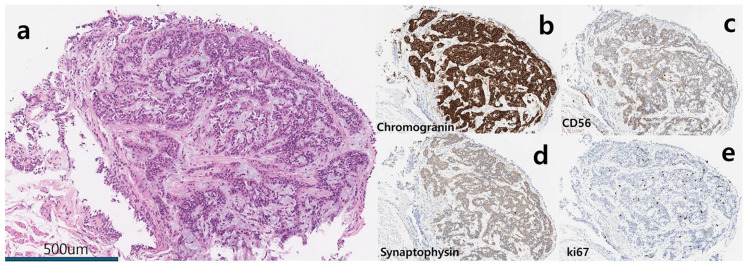
Pathologic findings of one of resected endobronchial nodules (×40). (**a**) A nodular lesion measuring approximately 1.5 mm showed neuroendocrine proliferation. Multiple papillary stalks with single-layered pseudostratified columnar cells with a relatively bland appearance were observed. Two mitoses per 2 mm^2^ were noted. (**b**–**d**) The nodule demonstrated positive immunohistochemical staining for chromogranin, CD56, and synaptophysin. (**e**) The Ki-67 index is approximately 6%. A diagnosis of carcinoid tumorlets was established. Pulmonary neuroendocrine proliferation can be divided into a spectrum of lesions that range from neuroendocrine cell hyperplasia to tumorlets and carcinoid tumors [1,2,3]. Carcinoid tumorlets are nodular neuroendocrine cell aggregates that extend beyond the basement membrane and measure 5 mm or less in size, with histomorphological features similar to carcinoid tumors [4]. Due to their small size, tumorlets are easily overlooked, leading to an underestimation of their true incidence. Tumorlets are typically located in the peripheral airways and appear as pulmonary micronodules on chest CT, resembling metastatic nodules or granulomas [5,6,7]. They are often discovered during histopathological examination of lung tissue resected for other reasons such as lung cancer [6]. Our case demonstrated bronchoscopic and CT findings of carcinoid tumorlets in the left main bronchus, along with corresponding pathological features. Reports of carcinoid tumorlets in the central airways are exceedingly rare, with only a few cases documented in the literature [8,9,10]. In our case, multiple tumorlets were observed five years after surgery for a typical bronchial carcinoid. Multicentric carcinoid tumors or tumorlets are not commonly encountered in clinical practice. However, a long-term observational study of 123 patients reported that approximately 12% of bronchial carcinoid cases exhibited multicentric manifestations, either as carcinoid tumors or tumorlets [11]. For patients with pulmonary carcinoids without distant metastases, surgery involving the complete removal of the primary tumor and a systematic lymph node dissection is recommended as the primary curative treatment. Pulmonary carcinoids may recur late, many years and even decades after treatment [2,12]. To detect late disease progression and recurrence, long-term follow-up using low dose chest CT is necessary [12]. Recurrence and poor prognosis are associated with atypical histology, lymph node metastases at diagnosis, presence of multiple carcinoid tumors or tumorlets, and genetic abnormalities, including large chromosomal alterations, specific gene mutations, and dysregulation of key oncogenic pathways [2,11,13]. The pathogenesis of multiple endobronchial tumorlets in this case remains uncertain. One possible explanation is recurrence of the previous typical carcinoid tumor through intramucosal spread, given that both the initial tumor and the subsequent tumorlets were in the left bronchus. Alternatively, they may represent a delayed manifestation of multicentric carcinoid tumors and tumorlets, supported by the established histogenetic relationship between these entities [11,12]. This case highlights an unusual presentation of carcinoid tumorlets in the central airways, emphasizing the importance of recognizing their potential for recurrence and atypical localization. It underscores the necessity for physicians to be aware that pulmonary neuroendocrine tumors can recur over the long term and may present in a multicentric fashion within the disease spectrum.
